# Repeated exposure affects susceptibility and responses of Atlantic salmon (*Salmo salar*) towards the ectoparasitic salmon lice (*Lepeophtheirus salmonis*)

**DOI:** 10.1017/S0031182023000847

**Published:** 2023-09

**Authors:** Mathias Stølen Ugelvik, Adele Mennerat, Stig Mæhle, Sussie Dalvin

**Affiliations:** 1 Institute of Marine Research, Bergen, Norway; 2 Department of Biological Sciences, University of Bergen, Bergen, Norway

**Keywords:** immune response, parasite success, repeated infections

## Abstract

Atlantic salmon (*Salmo salar*) is repeatedly exposed to and infected with ectoparasitic salmon lice (*Lepeophtheirus salmonis*) both in farms and in nature. However, this is not reflected in laboratory experiments where fish typically are infected only once. To investigate if a previous lice infection affects host response to subsequent infections, fish received 4 different experimental treatments; including 2 groups of fish that had previously been infected either with adult or infective salmon lice larvae (copepodids). Thereafter, fish in all treatment groups were infected with either a double or a single dose of copepodids originating from the same cohort. Fish were sampled when lice had developed into the chalimus, the pre-adult and the adult stage, respectively. Both the specific growth rate and cortisol levels (i.e. a proxy for stress) of the fish differed between treatments. Lice success (i.e. ability to infect and survive on the host) was higher in naïve than in previously infected fish (pre-adult stage). The expression of immune and wound healing transcripts in the skin also differed between treatments, and most noticeable was a higher upregulation early in the infection in the group previously infected with copepodids. However, later in the infection, the least upregulation was observed in this group, suggesting that previous exposure to salmon lice affects the response of Atlantic salmon towards subsequent lice infections.

## Graphical abstract

**Figure:**
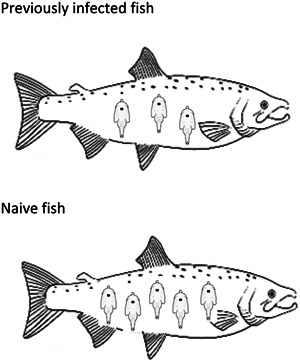

## Introduction

Parasites are ubiquitous in nature (Shaw *et al*., 1998) and a widespread feature of most populations is parasite aggregation, where few hosts carry high parasite loads while most hosts harbour few or none (Anderson and May, 1978; Anderson and Gordon, 1982; Shaw and Dobson, 1995; Shaw *et al*., 1998; Poulin, 2007). Although a significant part of this heterogeneity may be constrained by average infection levels and sampling effort (Poulin, 2013), explaining the remaining variation can be of considerable interest, especially for those host–parasite associations with high medical or economic importance. The most frequently invoked causes of heterogeneity in parasite loads are spatial and temporal variation in the distribution of infective stages (Bandilla *et al*., 2005; Poulin, 2013), including seasonal and climatic variation (Altizer *et al*., 2006; Lass and Ebert, 2006; Mennerat *et al*., 2021), but also variation in behaviour, condition or immunity of individual hosts (Lysne and Skorping, 2002; Wilson *et al*., 2002; Beldomenico and Begon, 2010; Sarabian *et al*., 2018).

Another factor contributing to parasite aggregation is the modulation of host immune responses by parasites once established on their hosts. Immune suppression by parasites resulting in higher parasite intensities, or immune stimulation reducing the levels of new infections can both affect the degree of parasite aggregation (Cox, 2001). The adaptive benefits of immune modulation for parasites likely depend on the characteristics of each host–parasite association. For sexual parasites typically found at low intensities, the facilitating effects of immune suppression may improve mating opportunities (Cox, 2001; Ugelvik *et al*., 2017*a*). Conversely, stimulation of immune responses against younger stages by established parasites may help limit the reproductive cost incurred by on-host competition (Selvan *et al*., 1993; Churcher *et al*., 2006; Hoffmann *et al*., 2016; Ugelvik *et al*., 2017*b*).

One way of investigating whether parasites stimulate or dampen host responses is to infect the same hosts repeatedly in laboratory conditions, where exposure is more homogenous than under natural conditions. An upregulation of immune responses should be reflected in a decrease in parasite loads across consecutive infections, while an increase in the success of repeated infections leading to parasite build-up would be expected if parasite suppress the immune responses of the host.

Here we address this question using the salmon louse (*Lepeophtheirus salmonis*), an ectoparasite of salmonid fishes. Infection with the parasite causes a well-described immune response in the skin also in susceptible species such as Atlantic salmon (*Salmo salar*) and rainbow trout (*Oncorhynchus mykiss*) (Braden *et al*., 2015; Dalvin *et al*., 2020; Ugelvik *et al*., 2022; Ugelvik and Dalvin, 2022; Øvergård *et al*., 2023). Salmon lice display aggregation on wild Atlantic salmon hosts in the northern Atlantic Ocean (Jacobsen and Gaard, 1997; Torrissen *et al*., 2013). The presence of several developmental stages on hosts caught at sea and the correlation between lice intensities and host age indicate that repeated infections occur naturally in this host–parasite system (Jacobsen and Gaard, 1997). According to an earlier study, hosts carrying adult lice from a previous infection acquire higher parasite loads, than naïve hosts, although the common garden nature of the experiment did not allow to exclude preferential settlement of parasites as a possible explanation. Intriguingly, the number of new lice was correlated with the number of adult lice already present on the fish, and this relationship vanished when the adult were removed from the fish prior to the second infection (Ugelvik *et al*., 2017*a*). One suggested explanation is that secretions produced by adult salmon lice may have immune regulatory effects (Fast *et al*., 2007; Øvergård *et al*., 2022), but the underlying changes in gene expression have not been established.

To test how previous exposure affects host responses to subsequent infection, Atlantic salmon with various infection histories were here experimentally exposed to infective salmon lice larvae (copepodids) and transcriptional changes, host weight, cortisol levels and parasite loads were assessed until the parasites reached the adult stage. In line with earlier findings, we expected fish carrying adult lice to acquire more lice than naïve fish. Also, salmon lice infection should cause a marked alteration in the expression of genes involved in the immune response and wound healing in the host skin, compared to non-infected control fish. Immune regulation should also be more pronounced in fish with a longer lasting infection (i.e. longer time for the parasite to modulate the hosts’ immune response) than in fish more recently infected. Finally, immune regulation by the louse should be more pronounced in fish that carried many (i.e. more lice modulating the hosts’ immune response), than in those that carried few lice.

## Materials and methods

### Study species

Salmon lice have a direct life cycle, consisting of 8 successively larger developmental stages (Hamre *et al*., 2013). The infection starts when copepodids attach to a host and develop through 2 sedentary stages (chalimi), then moulting into mobile pre-adults, before becoming sexually mature adult male or female lice (Hamre *et al*., 2013). The salmon louse feeds on host blood, skin and mucus and thereby causes damage to the skin (Grimnes and Jakobsen, 1996). This results in disturbances to the osmotic balance (Fjelldal *et al*., 2020), increases the likelihood of secondary infections (Nylund *et al*., 1994; Mustafa *et al*., 2000; Barker *et al*., 2019), reduces host body growth (Mennerat *et al*., 2012; Ugelvik *et al*., 2017*c*) and enhances host mortality at high lice intensities (Grimnes and Jakobsen, 1996; Fjelldal *et al*., 2020). Pathogenicity (i.e. the extent to which lice cause disease in the host) increases with parasite load but also depends on the developmental stage of the louse, with negative impacts becoming more evident as the lice develop into mobile pre-adults (Grimnes and Jakobsen, 1996; Fjelldal *et al*., 2020). This seems in part related to changes in gene expression (Eichner *et al*., 2018), exocrine gland development (Øvergård *et al*., 2016) and to the fact that bigger lice and especially adult females divert significant resources into egg production (Mennerat *et al*., 2012).

Salmon lice are generalists, i.e. they are found on multiple Atlantic (genera *Salmo* and *Salvelinus*) and Pacific Ocean (genus *Oncorhynchus*) salmonid species that differ considerably in their level of resistance against the parasite. These patterns are mostly attributed to interspecific variation in immunity amongst host species (Mackinnon, 1998; Jones *et al*., 2007; Braden *et al*., 2012, 2015), although they might also reflect genetic differentiation of the parasites between the Pacific and Atlantic Oceans (Skern-Mauritzen *et al*., 2014). The mechanisms conveying resistance to salmon lice also differ among host species. For instance, rapid rejection of the parasite in young pink salmon (*Oncorhynchus gorbuscha*) is mediated by proinflammatory responses (Jones *et al*., 2007), while resistance in Coho salmon (*Oncorhynchus kisutch*) is associated with epithelial hyperplasia (Johnson and Albright, 1992). Amongst salmonid host species, Atlantic salmon is the highly susceptible to salmon lice (Braden *et al*., 2015). Within this host species, differences in resistance among families indicate genetic variation for this trait (Glover *et al*., 2005; Kolstad, 2005; Gjerde *et al*., 2011). Immune suppression in the host was previously suggested (Fast *et al*., 2007; Øvergård *et al*., 2016; Braden *et al*., 2020), but the underlying mechanisms remain unknown (Eichner *et al*., 2015; Hamilton *et al*., 2018; Dalvin *et al*., 2021).

### Experimental set-up

#### General principle

At the start of the experiment, 320 Atlantic salmon from the same cohort (Aquagen strain) were sedated and tagged with a passive integrated transponder tag (PIT tag). Their weight was recorded [217 g (±1.5 s.e.)] before the fish were randomly divided into 16 600 L tanks (4 replicate tanks per treatment). Tanks were arbitrarily assigned to either of the 4 treatment groups differing in their infection history (naïve or previously infected hosts; see details below). These 4 treatment groups were then exposed to infective salmon lice larvae (copepodids) from a common pool and the development of parasites monitored. Throughout the experiment, fish were maintained in 12°C sea water, 35 ppt salinity. Fish were sedated by carefully netting them into a bucket containing 15 mg L^−1^ Finquel (tricaine mesylate) in 10 L of sea water. Parasite load was recorded on the sampled fish at the chalimus, pre-adult and adult stage (sampling I, II and III at 11, 20 and 30 days post-exposure, respectively). At each sampling, the fish were weighed, and blood and skin samples were taken to measure plasma cortisol levels (as a measure of stress levels) and immune and wound healing gene transcription, respectively.

#### Treatment groups

The purpose of the study was specifically to describe how previous exposure affects host responses to subsequent infection. Hence, groups of fish (80 individuals) were infected in 3 different ways and compared to fish infected only once with a single dose of infective copepodids (60 copepodids per fish), thereafter referred to as the ‘Cop’ group. Fish in the second group (thereafter ‘Copx2’) were infected once with a double dose of infective copepodids (120 copepodids per fish). In the third group (thereafter ‘Copcop’), fish were infected twice with a single dose (60 copepodids per fish), with a 30-day interval between the 2 exposures. Lastly, the fourth group (thereafter ‘Aducop’ group) adult lice were first placed on the fish (4 females and 3 males per fish), and 7 days later, these fish were exposed to a single infective dose (60 copepodids per fish).

#### Infection procedure

The copepodids and adult lice used in the experiment were collected from naturally infected Atlantic salmon held at a commercial fish farm in Masfjorden (Norway) and kept in the laboratory in seawater at 12°C at 35 ppt salinity for 1 generation prior to the infection. All infections with copepodids were done by lowering the water level to one-third of its volume and adding infective salmon lice copepodids to the tanks. The inflow of water was maintained (12 L min^1^), but the outlet was blocked until normal water level was restored. Only fish in the Copcop group were infected with copepodids in the first infection, while the other 3 treatment groups were sham infected (i.e. treated similarly to the Copcop group, but no copepodids were poured into the water).

Seven days prior to the second infection, all fish were individually sedated, identified by the PIT tag and their weights recorded. In the Copcop group, the parasite loads resulting from the first infection were also recorded. In the Aducop groups, adult lice reared on another batch of Atlantic salmon were carefully placed directly onto the fish. All fish were returned to their respective tanks. Finally, 30 days after the first infection of fish in the Copcop group, fish belonging to all treatment groups were infected either with a double (Copx2) or a single dose (Cop, Copcop and Aducop groups) of copepodids from the same cohort.

### Sampling procedure

For each sampling, 5 fish from each tank (in total 20 fish per treatment group) were carefully netted and sedated, the PIT tag was read, number of lice enumerated and fish weight was recorded. The fish was subsequently humanely euthanized with a sharp blow to the head and a blood sample was taken. Blood samples were kept on ice until centrifugation and plasma collection. Plasma was stored at −80°C until analysis. Plasma cortisol concentration was determined using an ELISA assay kit (IBL International GmbH) with a Sunrise microplate reader (Tecan, Switzerland). Furthermore, 2 skin samples from the flank of each fish were taken immediately after euthanization: one from directly underneath the louse (lice-positive sample), and the other from a similar location without louse (lice-negative sample). Skin samples were frozen at −80°C in 1.5 mL PreMax™-plate tubes containing 2 stainless-steel beads (Nerliens Meszansky) for later RNA extraction.

### RNA purification

A volume of 500 μL Tri reagent (Sigma Aldrich) was added to the 1.5 mL PreMax™-plate tubes containing skin samples and homogenized for 2 min at 1400 rpm (FastPrep 96, MP Biomedicals). Thereafter, samples were kept at room temperature for 5 min before adding 100 μL chloroform (Sigma Aldrich), then vortexed for 1 min at 1400 rpm (FastPrep 96, MP Biomedicals) and centrifuged at 16 000 rcf at 4°C for 15 min. A volume of 200 μL supernatant was withdrawn and 400 μL RLT (Qiagen) and 600 μL 70% ethanol was added. RNA was further extracted following the RNeasy-Micro protocol (Qiagen). Quality and quantity of RNA were assessed with a NanoDrop™-1000 spectrophotometer (NanoDrop Technologies, ThermoFisher Scientific) and purified RNA was stored at –80°C until further use.

### cDNA

Reverse transcription was carried out using SuperScript^®^ VILO™ complementary DNA (cDNA) synthesis kit (ThermoFisher Scientific) according to manufacturer recommendations in a total volume of 10 μL along with the negative control (RTneg) and no template control (NTC). The samples were diluted with nuclease-free, sterile water (VWR) to get an RNA concentration of 300 ng μL^−1^, and 4 μL RNA was transferred and mixed with 6 μL Vilo™ cDNA synthesis mix (containing 3 μL nuclease-free, sterile water, 2 μL 5XVilo™ reaction mix and 1 μL 10× superscript^®^ enzyme mix) in a total of 1200 ng μL^−1^. The RTneg was prepared by replacing the 10× superscript^®^ enzyme mix with nuclease-free, sterile water, while only nuclease-free, sterile water was pipetted into the NTC wells.

Samples were incubated following the manufacturer's instruction first at 25°C for 10 min, thereafter at 42°C for 60 min, before the reaction was terminated at 85°C for 5 min. Samples were frozen at −20°C and cDNA was later diluted (1:20) by mixing 95 μL nuclease-free, sterile water and 5 μL of cDNA prior to the real-time qPCR assay.

### RT-qPCR

Reverse transcription-quantitative polymerase chain reaction (RT-qPCR) was performed in QuantStudio™5 system (ThermoFisher Scientific). Assays were run in 7 μL reactions, including 3.5 μL master mix (BrilliantIII ultra-fast SYBR^®^green qPCR master mix, Agilent), 0.28 μL of forward primer, 0.28 μL of reverse primer, 0.10 μL reference dye (1:500), 0.84 μL nuclease-free, sterile water and 2 μL template in a total quantity of 12 ng cDNA. qPCR cycling conditions were 95°C for 3 min, then 40 cycles of 95°C for 5 s and 60°C for 20 s. The melt curve stage had a denaturation step at 95°C, annealing step at 60°C and a dissociation step at 95°C.

All assays were tested using both negative control (RTneg) and no template control (NTC). Analyses of messenger RNA (mRNA) levels were conducted using the simplified 2^−ΔΔCt^ method as used by Dalvin *et al*. (2020). The proposed function, the role of the selected transcripts on host responses towards parasite and primers used are found in Tables 1 and 2. Elongation factor 1-*α* (EF1-*α*) and receptor-like protein 1 were used as reference genes. Results are presented as fold change in the different treatment groups at each lice stage (both negative and positive samples) compared to lice-negative samples in the Cop group at the same development stage. Changes in threshold cycle (ΔCt) value were calculated as differences between RNA levels of the gene of interest and the arithmetic mean of the reference genes. ΔΔCt was quantified as the difference between ΔCt in the Cop (positive), Copx2, Copcop and Aducop (lice-negative and -positive) compared to the average ΔCt of lice-negative samples from the Cop group. Only expressional differences between groups with a minimum of 2-fold differences in mRNA and *P* < 0.05 were considered significant.

**Table 1.** Investigated transcripts, main function and previously reported expression in salmon lice-infected fish

Gene	Function	Involvement in salmon lice infection
Immunoglobulin T	Fish-specific immunoglobulin found on mucosal surfaces to protect against infections (Hordvik, 2015)	Upregulated early in the skin of lice-infected Atlantic salmon (Tadiso *et al*., 2011), but downregulated in infected rainbow trout (Dalvin *et al*., 2020)
Immunoglobulin M	Important for both innate and adaptive immune responses (Mashoof and Criscitiello, 2016)	Reduced transcription at the site of lice attachment in susceptible species such as rainbow trout, sockeye and Atlantic salmon (Braden *et al*., 2015; Dalvin *et al*., 2020)
Interleukin 1-*β*	A multifunctional cytokine produced by stimulated cells and regulates immune responses and lysozyme activity (Secombes *et al*., 2001; Zou and Secombes, 2016)	Higher transcription in lice-infected salmonids (Braden *et al*., 2012; Braden *et al*., 2015; Øvergård *et al*., 2018)
Interleukin 2	Produced by T-cells and increases proliferation of immune cells, enhances phagocytosis and regulates both anti- and proinflammatory responses (Wang *et al*., 2018)	A role in host responses towards salmon lice not previously investigated
Interleukin 4/13a	Linked to activation of anti-inflammatory macrophages by Th-2, eosinophils, basophils and natural killer T-cells (Luckheeram *et al*., 2012; Grayfer *et al*., 2018)	Increased expression at the site of lice attachment in lice-infected salmonids, including Atlantic salmon (Braden *et al*., 2015; Øvergård *et al*., 2018; Dalvin *et al*., 2020)
Interleukin 8	Associated with macrophages and leucocytes and mediates the migration of immune cells to the site of infection (Covello *et al*., 2009; Brunner *et al*., 2020)	Increased transcription at the site of lice attachment in Atlantic, coho and sockeye salmon (Braden *et al*., 2015; Øvergård *et al*., 2018)
Interleukin 10	Multifunctional cytokine-regulating immune response by inhibiting differentiation of monocytes and reduces phagocytosis and suppresses the expression of MH class II molecules and proinflammatory cytokines (Chaudhry *et al*., 2011)	Higher expression in lice infection-resistant coho salmon, but not in Atlantic salmon (Braden *et al*., 2015)
Cathelicidin 2	Antimicrobial peptide that increases phagocytosis, influences innate immunity and enhances interleukin level in peripheral blood leucocytes (Brunner *et al*., 2020)	Increased expression at the site of lice attachment observed in rainbow trout, Atlantic and sockeye salmon (Braden *et al*., 2015; Dalvin *et al*., 2020)
Inducible nitric oxide synthase	Marker of activated M1 macrophages and involved in the production of nitric oxide (Grayfer *et al*., 2018)	Upregulated in Atlantic salmon infected with adult lice, while reduced expression is observed at the site of lice attachment in rainbow trout (Braden *et al*., 2015; Dalvin *et al*., 2020)
Serum amyloid A	Produced by stimulated hepatic cells and affects pathogens, acts as opsonin, activates the complement system and influences the production of cytokines (Kania *et al*., 2014; Grayfer *et al*., 2018)	Associated with lice resistance, which is corroborated by higher expression in resistant than in susceptible salmonids (Braden *et al*., 2015; Dalvin *et al*., 2020)
Complement component 3	Found in fish serum and involved in opsonization and lysis of foreign cells (Holland and Lambris, 2002; Grayfer *et al*., 2018)	Downregulated at the site of lice attachment in rainbow trout (Dalvin *et al*., 2020) and in infected Atlantic salmon (Holm *et al*., 2015)
Tumour necrosis factor-*α*	Proinflammatory cytokine regulating other cytokines, involved in proliferation and differentiation of macrophages, recruits immune cells to the site of infection and is involved in apoptosis (Hong *et al*., 2013; Grayfer *et al*., 2018)	A role in resistance against lice and involvement in wound healing is proposed (Braden *et al*., 2015; Holm *et al*., 2015)
Transforming growth factor-*β*	Promotes proinflammatory responses early in the infection, while later suppressing inflammation and is involved in regulating the extracellular matrix (Ignotz and Massagué, 1986; Qi *et al*., 2016)	Upregulated transcription in lice-damaged skin (Skugor *et al*., 2008), while it was downregulated in another study on Atlantic salmon (Holm *et al*., 2015). Increased levels in resistant species in response to lice infections (Braden *et al*., 2015)
Collagen 10-α*	Collagens are crucial for the extracellular matrix and important for wound healing and tissue remodelling (Castillo-Briceño *et al*., 2011)	Reduced transcription in the skin of lice-infected Atlantic salmon (Skugor *et al*., 2008)
Fibronectin precursor*	Fibronectin increases migration of fibroblasts and epidermal cells and affects adhesion and repair of damaged tissue (Valenick *et al*., 2005)	Downregulated in the skin of infected Atlantic salmon both late and early in the infection (Skugor *et al*., 2008)
Matrix metalloproteinase 9*	Important for tissue remodelling and dysregulation is associated with the presence of chronic wounds in fish (Skugor *et al*., 2008; Braden *et al*., 2012; Braden *et al*., 2020)	Activated in lice-infected fish in several salmonids including Atlantic salmon (Braden *et al*., 2015)
Cluster of differentiation 8-*α*	A marker for cytotoxic cells that are important for removal of virus-infected cells (Somamoto *et al*., 2014)	Reduced transcription at the site of lice attachment in rainbow trout, coho, sockeye and Atlantic salmon (Braden *et al*., 2015; Dalvin *et al*., 2020)
Cluster of differentiation 4	A marker of T-helper cells and is involved in regulating cellular and humoral immune responses (Chaplin, 2010; Luckheeram *et al*., 2012)	Upregulated in Atlantic salmon infected with salmon lice (Skugor *et al*., 2008; Tadiso *et al*., 2011), but not in rainbow trout (Dalvin *et al*., 2020)

*Denotes genes involved in wound healing.

**Table 2.** Forward (F) and reverse (R) primers for the investigated immune and wound healing genes (denoted*) used in RT-qPCR setup

Gene	Primers	Accession number	Amplicon length
Immunoglobulin T	F:GGTGGTCATGGACGTACTATTT		
	R:CCTGTGCAGGCTCATATCTT	GQ 907004.1	98
Immunoglobulin M	F:TGAGGAGAACTGTGGGCTACACT		
	R:TTAATGACTACTGAATGTGCAT	S48652	67
Interleukin 8	F:GCATCAGAATGTCAGCCAGCC		
	R:ACGCCTCTCAGACTCATCCC	NM_001140710.2	79
Interleukin 1-*β*	F:GCTGGAGAGTGCTGTGGAAGA		
	R:TGCTTCCCTCCTGCTCGTAG	NM_001123582.1	73
Interleukin 4/13a	F:CGTACCGGCAGCATAAAAATCACCATTCC		
	R: CCTTGCATTTTGTGGTGGTCCCA	NM_001204895	147
Interleukin 10	F:GCTATGGACAGCATCCTGAAGTT		
	R:GGTTGTTCTGCGTTCTGTTGTT	EF165028.1	76
Cathelicidin 2	F: ACACCCTCAACACTGACC		
	R: CCTCTTCTTGTCCGAATCTTCT	AY542961.1	382
Cluster of differentiation 8-*α*	F:TAGAGTGCAAGACAACGCTGGAATGGA		
	R:TCTCGAGCCTTTTTGAAAGCCTTCAG	NM_001123583.1	150
Tumour necrosis factor-*α*	F: AGGTTGGCTATGGAGGCTGT		
	R:TCTGCTTCAATGTATGGTGGG	NM_001123589	173
Cluster of differentiation 4	F:GTGGAGGTGCTACAGGTGTTTTC R:GGGGAGGAGCCTAAAGCG	EU409794.1	158
Elongation factor 1-*α*	F:CACCACCGGCCATCTGATCTACAA		
	R:TCAGCAGCCTCCTTCTCGAACTTC	NM_001123629.1	78
Complement component 3	F:TCCCTGGTGGTCACCAGTACAC		
	R:ATGATGGTGGACTGTGTGGATC	BI468074	157
Serum amyloid A	F:AGCTGCTCGAGGTGCTAAAG		
	R:ATGTCCTCGACCACTGGAAC	NM_001146565.1	193
Collagen 10-α*	F:TGGTGCTCTTTGACTGCCTGTAA		
	R:CATCCTGTGTGTTGCAATATCACA	EG837148	180
Fibronectin precursor*	F:GCATGTCTGAGACGGGCTTCAA		
	R: AGTCACATCGGAAGTGTCCACTGC	XM_014123350.2	72
Matrix metalloproteinase 9*	F:AGTCTACGGTAGCAGCAATGAAGGC		
	R:CGTCAAAGGTCTGGTAGGAGCGTAT	NM_001140457.1	72
Transforming growth factor-*β*	F:AATCGGAGAGTTGCTGTGTGCGA		
	R:GGGTTGTGGTGCTTATACAGAGCCA	EU082211	178
Receptor-like protein 1	F: ACTATGGCTGTCGAGAAGGTGCT		
	R:TGTACTCGAACAGTCGTGGGTCA	CB516726	118
Interleukin 2	F:GCGGATGTAGAGAAAAGCATTG R:CATTCTGACGAGTCCGTTCTGAT	HE805272.1	155
Inducible nitric oxide synthase	F:GGAGAGCCTTCTGGTTG		
	R:ACCTTAACTTGTTCCTGAGATAC	AF 088999.1	116

### Statistical analyses

All analyses were performed using the statistical program environment R 4.3.1 (R core team, 2022). For all models, normality and heteroscedasticity of residuals were performed by visual inspection.

### Parasite success

The effect of treatment (Cop, Copx2, Copcop and Aducop) on lice infection success and subsequent survival was investigated with a generalized linear model (glm) fitted with quasibinomial distribution. The model included the proportion of lice that settled and survived on the fish (thereafter ‘parasite success’) as a binomial response variable and treatment as a predictor variable. The binominal predictor variable combined (using the cbind function) the number of lice that successfully infected and survived on the host and those that did not. Two models were fitted to investigate how the treatment affected parasite success up until the chalimus and pre-adult stage, respectively. *Post hoc* comparisons between treatments were done using the emmeans function in R.

### Host stress response

The effect of treatment on host stress level for each stage (chalimus, pre-adult and adult) was tested by fitting a linear mixed-effect model (lme) with cortisol concentration as response variable, and treatment and parasite load (total number of lice on the fish) as predictor variables. Tank was defined as a random-effect factor. *Post hoc* comparisons between treatment groups were done using analysis of variance (ANOVA) and a Tukey's *post hoc* test.

### Fish-specific growth rate

To test whether the initial weight of the hosts differed across treatments, a linear mixed-effect (lme) model was fitted with start weight as response variable, treatment as predictor variable and tank as a random effect. The effects of treatment and parasite load on host growth were investigated with a linear mixed-effect model (lme) at each parasite stage (chalimus, pre-adult and adult stage, respectively). The response variable was the specific growth rate of the host as in Fjelldal *et al*. (2020), and treatment and parasite load were included as predictor variables. Tank was defined as a random-effect factor. *Post hoc* comparisons between treatment groups were done using ANOVA and a Tukey's *post hoc* test.

## Results

### Parasite success

**Figure 1.**
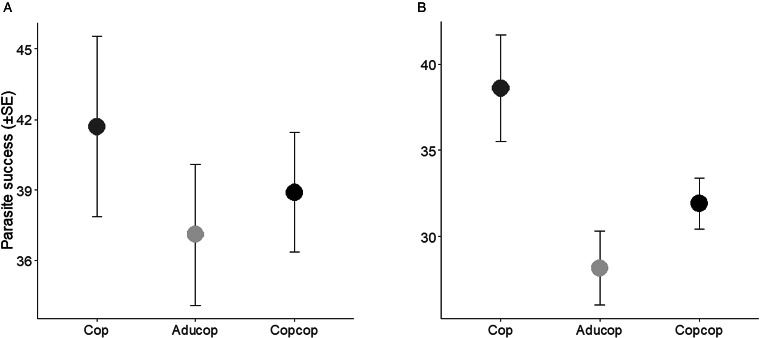
Parasite success (the number of lice infecting and surviving on the host until sampling) for the treatment groups infected with a single dose of copepodids (60 lice per fish) at the chalimus (a) and pre-adult stages (b), respectively. Fish in the Aducop group were previously infected with adult lice (4 females and 3 males per fish), while fish in the Copcop group was previously infected with copepodids (60 lice per fish × 2). Fish in the Cop group was unexposed to salmon lice prior to the copepodid infection.

**Figure 2.**
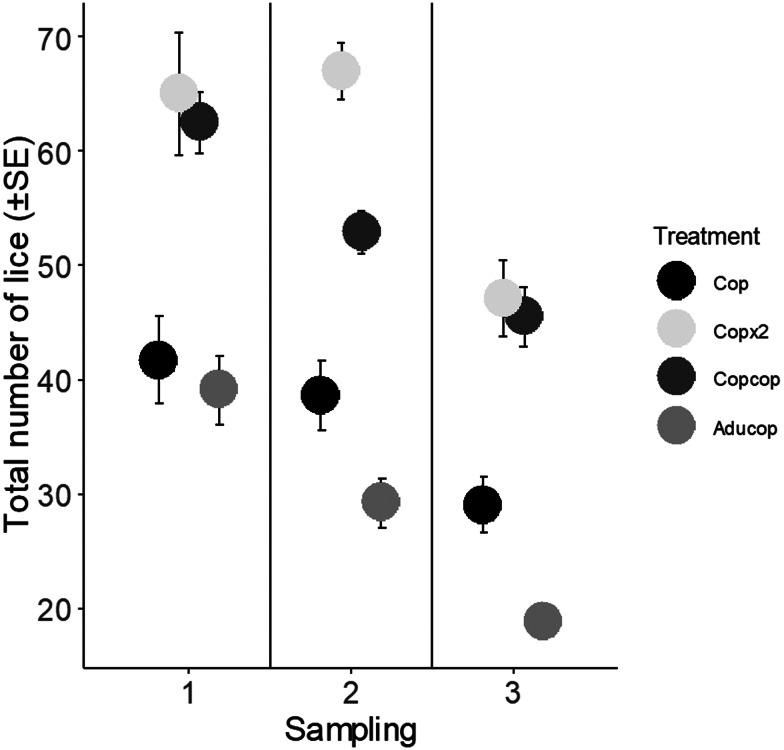
Total number of lice (i.e. lice from both infections for fish in the Aducop and Copcop groups) (±s.e.) for the different treatments (Aducop, Copcop, Cop and Copx2 groups) depending on sampling [1 (chalimus), 2 (pre-adult) and 3 (adult)].

At the first sampling (i.e. lice had become chalimus), lice success (i.e. the ability to infecte and survive on the host until sampling) did not differ between the treatment groups (Fig. 1a, Tables 3 and 4, Supplementary Table 2). At the second sampling (i.e. lice had devloped into pre-adults), lice were more sucessful on fish in the Cop group than in the fish with prior exposure to adult salmon lice [*P* = 0.001 (glm)] (Fig. 1b, Tables 3 and 4, Supplementary Table 2). There were no differences between the other treatments. Total number of lice (i.e. lice from previous and new infection) was highest in fish exposed to a double dose of copepodids (Copx2 and Copcop groups) (Fig. 2, Supplementary Table 2).

**Table 3.** Output from glm models exploring the effect of treatment on lice success (i.e. infection success and survival) and at the chalimus and pre-adult stages

	Estimate	s.e.	*T*-value	*P* value	Stage
Intercept (Aducop)	0.46	0.2	2.3	0.027	Chalimus
Treatment Cop	0.15	0.29	0.5	0.6	
Treatment Copcop	0.28	0.29	0.94	0.35	
Treatment Copx2	−0.31	0.28	−1.1	0.27	
Intercept (Aducop)	−0.12	0.13	−0.93	0.35	Pre-adult
Treatment Cop	0.64	0.19	3.42	0.001*	
Treatment Copcop	0.25	0.18	1.35	0.18	
Treatment Copx2	0.36	0.19	1.9	0.058	

* Indicates significant differences between the treatments.

**Table 4.** To investigate if lice success differed between treatments, multiple comparisons were performed using the emmeans function in R at both the chalimus and pre-adult stages

Contrast	Estimate	s.e.	*Z*-ratio	*P* value	Stage
Aducop-Copcop	−0.15	0.29	−0.53	0.95	Chalimus
Aducop-Cop	−0.27	0.29	−0.94	0.78	
Aducop-Copx2	0.31	0.28	1.11	0.69	
Copcop-Cop	−0.12	0.30	−0.415	0.97	
Copcop-Copx2	0.47	0.29	1.63	0.36	
Cop-Copx2	0.59	0.29	2.04	0.18	
Aducop-Cop	−0.64	0.18	−3.42	0.0035*	Pre-adult
Aducop-Copcop	−0.25	0.19	−1.35	0.53	
Aducop-Copx2	−0.36	0.19	−1.92	0.22	
Cop-Copcop	0.395	0.19	2.12	0.15	
Cop-Copx2	0.287	0.19	1.54	0.41	
Copcop-Copx2	−0.108	0.18	−0.59	0.94	

*Indicates significant differences between the treatments.

### Host stress response

**Figure 3.**
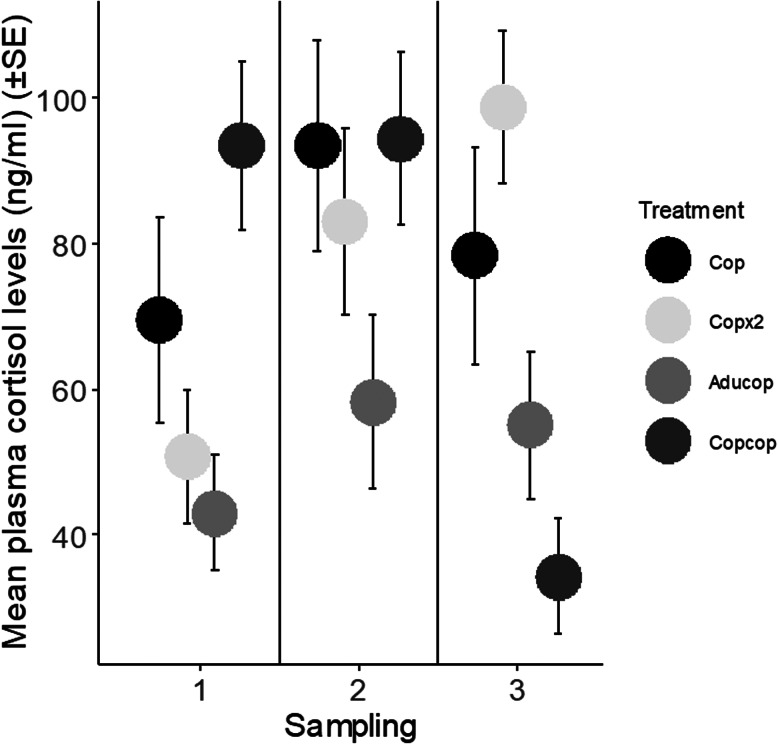
Mean plasma cortisol levels (ng mL^−1^) (±s.e.) in fish depending on sampling (1–3) for the different experimental treatments. Fish belonging to the Aducop group was previously infected with adult lice (4 females and 3 males per fish), while fish in the Copcop group was previously infected with (60 copepodids per fish). Fish in the Cop and Copx2 group were unexposed to salmon lice prior to the copepodid infection. During the infection, fish in the Cop, Aducop and Copcop groups were infected with a single dose of copepodids (60 copepodids per fish), while fish in the Copx2 group was infected with a double dose of copepodids (120 copepodids per fish).

At the chalimus stage, cortisol levels (a proxy for stress) were higher in fish from the Copcop group than in the Aducop and Copx2 groups (Tukey's *post hoc* test, *P* = 0.007 and 0.003, respectively). At the pre-adult stage, cortisol levels were not affected by either treatment or number of lice (Tables 5 and 6). At the adult stage, mean cortisol levels were lower in the Copcop group than in the Cop and Copx2 groups (Tukey's *post hoc* test, *P* = 0.03 and *P* < 0.001). Additionally, it was higher in the Copx2 than in the Aducop group (Tukey's *post hoc* test, *P* = 0.003) (Tables 5 and 6, Fig. 3).

**Table 5.** Output from linear mixed-effect model exploring the effect of the total number of lice and treatment on plasma cortisol levels depending on stage

	Value	s.e.	d.f.	*T*-value	*P* value	Stage
Intercept (Aducop)	63	38.8	59	1.6	0.1	Chalimus
Treatment Cop	−18.98	50.2	12	−0.37	0.7	
Treatment Copcop	81.04	63.7	12	1.27	0.23	
Treatment Copx2	5.96	52.17	12	0.11	0.91	
Total number of lice	−0.52	0.87	59	−0.59	0.55	
Treatment Cop ×total number of lice	1.1	1.06	59	1.04	0.3	
Treatment Copcop × total number of lice	−0.3	1.15	59	−0.27	0.79	
Treatment Copx2 × total number of lice	0.23	0.98	59	0.23	0.81	
Intercept (Aducop)	63.39	48.5	59	1.3	0.2	Pre-adult
Treatment Cop	19.74	65.6	12	0.30	0.76	
Treatment Copcop	−2.50	91.6	12	−0.03	0.98	
Treatment Copx2	−155.5	96.50	12	−1.6	0.13	
Total number of lice	−0.16	1.57	59	−0.1	0.92	
Treatment Cop × total number of lice	0.42	1.89	59	0.22	0.82	
Treatment Copcop × total number of lice	0.79	2.13	59	0.37	0.7	
Treatment Copx2 × total number of lice	2.77	1.99	59	1.39	0.17	
Intercept (Aducop)	90	41.45	58	2.17	0.03	Adult
Treatment Cop	−51.98	56.28	12	−0.9	0.37	
Treatment Copcop	−104	59.66	12	−1.75	0.10	
Treatment Copx2	20.5	54.9	12	0.37	0.7	
Total number of lice	−1.85	1.98	58	−0.93	0.35	
Treatment Cop × total number of lice	3.28	2.28	58	1.44	0.16	
Treatment Copcop × total number of lice	2.9	2.15	58	1.4	0.18	
Treatment Copx2 × total number of lice	1.5	2.1	58	0.72	0.47

**Table 6.** Output from ANOVA test exploring the effect of the total number of lice and treatment on plasma cortisol levels depending on stage

	d.f.	SumSQ	MeanSQ	*F*-value	Pr(>*F*)	Stage
Treatment	3	30301	10100	4.50	0.006*	Chalimus
Total number of lice	1	1417	1417	0.631	0.43	
Treatment × total number of lice	3	14954	4985	2.22	0.09	
Treatment	3	16431	5477	1.719	0.17	Pre-adult
Total number of lice	1	4722	4722	1.48	0.23	
Treatment × total number of lice	3	15197	5066	1.59	0.20	
Treatment	3	45914	15305	6.77	0.0004*	Adult
Total number of lice	1	957	957	0.42	0.52	
Treatment × total number of lice	3	18866	6289	2.78	0.04*	

* Indicates significant differences between the treatments.

### Fish-specific growth rate

**Figure 4.**
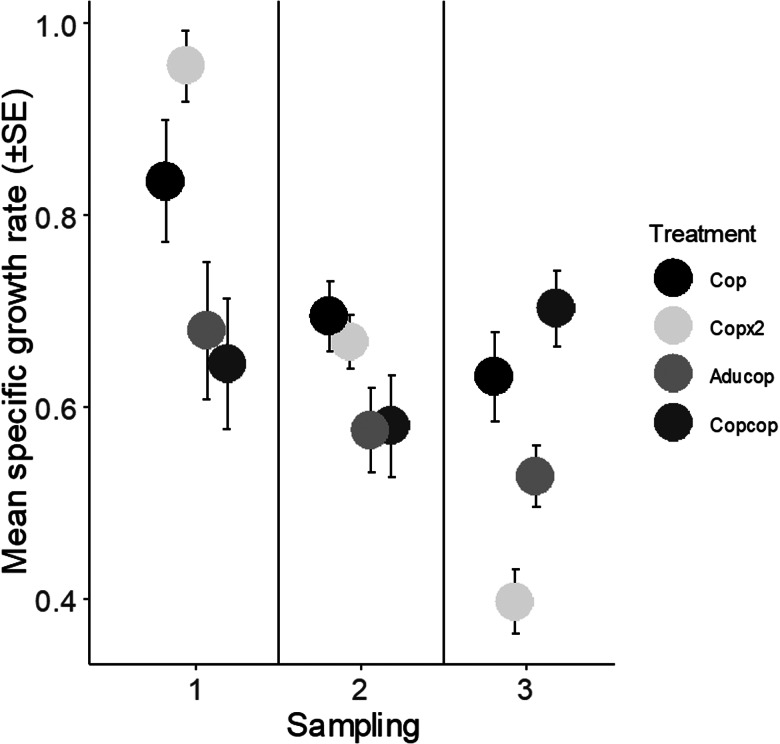
Mean specific growth rate (±s.e.) at sampling 1–3. Start weight for all groups was recorded at 24 days past the first exposure for the Copcop group (lice from the previous infection had become pre-adults). At this date, fish belonging to the Aducop group were also infected with adult lice (4 females and 3 males per fish). Seven days later (30 dpi), all groups were infected with either a single (Aducop, Copcop and Cop) or a double dose (Copx2) of infectious copepodids (60 and 120 lice per fish, respectively). Weight was recorded when louse from infection had developed into the chalimus (sampling 1, 42 dpi), pre-adult (sampling 2, 51 dpi) and adult stage (sampling 3, 60 dpi).

There was no significant difference in start weight between the treatments (ANOVA, *P* = 0.92, Tables 7 and 8), and overall, the effect of treatment and number of lice on fish weight was small. However, at the chalimus stage, the specific growth rate was affected by the total number of lice (lme, *P* = 0.002) and was higher in the Copx2 group than in the Copcop and Aducop groups (Tukey's *post hoc* test, *P* = 0.001 and *P* = 0.0065) (Fig. 4, Tables 9 and 10). At the pre-adult stage, the growth rate was not affected by either the number of lice or treatment, but at the adult stage, the growth rate was higher in the Copcop group than in the Aducop and Copx2 groups (Tukey's *post hoc* test, *P* = 0.009 and *P* < 0.001, respectively). The growth rate was also higher in the Cop group than in the Copx2 group (Tukey's *post hoc* test, *P* = 0.0003) (Fig. 4, Tables 9 and 10).

**Table 7.** Output from the linear mixed-effect model to explore if start weight differed between the treatments

	Value	s.e.	d.f.	*T*-value	*P* value
Intercept (Aducop)	217	3.13	222	69.43	<0.001
Cop	1.08	4.42	12	0.24	0.81
Copcop	−0.1	4.4	12	−0.02	0.98
Copx2	−1.93	4.4	12	−0.44	0.67

**Table 8.** Output from the ANOVA test to explore if start weight differed between the treatments

	d.f.	SumSQ	MeanSQ	Pr(>*F*)
Treatment	3	280	93.4	0.92

**Table 9.** Output from the linear mixed-effect model exploring the effect of treatment and total number of lice on specific weight gain depending on stage

	Value	s.e.	d.f.	*T*-value	*P* value	Stage
Intercept (Aducop)	0.23	0.18	60	1.23	0.21	Chalimus
Treatment Cop	0.36	0.24	12	1.5	0.15	
Treatment Copcop	−0.20	0.28	12	−0.7	0.50	
Treatment Copx2	0.47	0.25	12	1.8	0.08	
Total number of lice	0.11	0.003	60	3.23	0.002*	
Treatment Cop × total number of lice	−0.005	0.004	60	−1.38	0.17	
Treatment Copcop × total number of lice	−0.0001	0.004	60	−0.30	0.76	
Treatment Copx2 × total number of lice	−0.008	0.004	60	−1.9	0.06	
Intercept (Aducop)	0.40	0.16	59	2.47	0.06	Pre-adult
Treatment Cop	0.09	0.23	12	0.40	0.69	
Treatment Copcop	0.29	0.27	12	1.11	0.29	
Treatment Copx2	0.27	0.30	12	0.91	0.38	
Total number of lice	0.006	0.004	59	1.24	0.22	
Treatment Cop × total number of lice	−0.001	0.006	59	−0.17	0.86	
Treatment Copcop × total number of lice	−0.008	0.006	59	−1.39	0.18	
Treatment Copx2 × total number of lice	−0.006	0.006	59	−1.03	0.30	
Intercept (Aducop)	0.44	0.15	59	2.92	0.005	Adult
Treatment Cop	0.06	0.2	12	0.27	0.79	
Treatment Copcop	0.18	0.2	12	0.88	0.41	
Treatment Copx2	−0.13	0.2	12	−0.65	0.53	
Total number of lice	0.004	0.007	59	0.63	0.53	
Treatment Cop × total number of lice	0.001	0.008	59	0.006	0.99	
Treatment Copcop × total number of lice	−0.003	0.007	59	−0.36	0.72	
Treatment Copx2 × total number of lice	−0.0029	0.007	59	−0.389	0.69	

* Indicates significant differences.

**Table 10.** Output from the ANOVA test exploring if the specific growth rate was affected by treatment and the total number of lice

	d.f.	SumSQ	MeanSQ	*F*-value	Pr(>*F*)	Stage
Treatment	3	1.20	0.41	6.2	<0.001*	Chalimus
Total number of lice	1	0.57	0.56	8.5	0.004*	
Treatment × total number of lice	3	0.13	0.1	1.56	0.21	
Treatment	3	0.22	0.07	2.16	0.1	Pre-adult
Total number of lice	1	0.0009	0.0009	0.03	0.873	
Treatment × total number of lice	3	0.13	0.04	1.27	0.29	
Treatment	3	1.05	0.35	12	<0.001*	Adult
Total number of lice	1	0.003	0.03	1.04	0.312	
Treatment × total number of lice	3	0.08	0.03	0.9	0.436	

* Indicates significant differences.

### Transcriptional responses in the skin of the host

**Figure 5:**
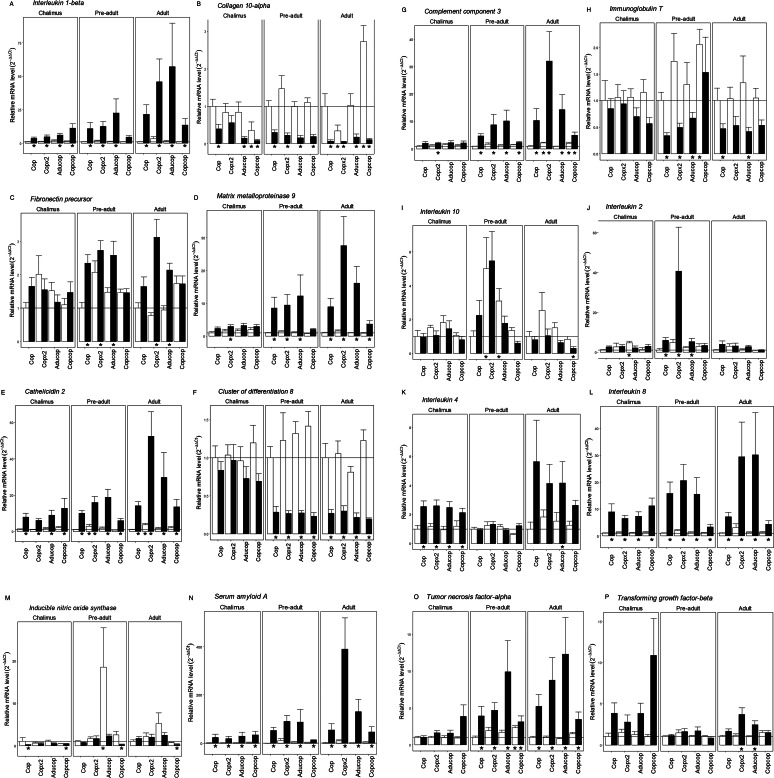
(a–p). Relative mRNA levels (±s.e.) in skin samples for selected immune and wound healing gene transcripts depending on treatment and lice development stage in infected fish (Cop, infected once with a single dose of copepodids; Copx2, infected once with a double dose of copepodids; Copcop, infected twice with a single dose of copepodids; Aducop, infected with adult lice and later infected with a single dose of copepodids). Skin samples were taken either directly under the lice (black columns) or away from the site of infection (white columns). The expression level is expressed as 2^−ΔΔCT^ (±s.e.) in sites under and away from site of infection compared to sites without lice in the Cop group (*denotes significant difference in expression). The horizontal line is set at *y* = 1.

At the chalimus stage, there was an upregulation of cytokines in the skin at the site of lice attachment in all treatment groups; this included interleukin 1-*β* (Fig. 5a), interleukin 4 (Fig. 5k) and interleukin 8 (Fig. 5l). For both interleukin 1-*β* and interleukin 8, the highest upregulation was seen in the Copcop group. Interleukin 2 was upregulated in non-lice sites in the Aducop group only (Fig. 5j). Higher transcription at the site of lice attachment was evident in the acute-phase protein serum amyloid A (Fig. 5n) and the antimicrobial peptide cathelicidin 2 (Fig. 5e). The expression of these transcripts was highest in previously infected fish (Copcop and Aducop groups). Whereas complement component 3 (Fig. 5g) and matrix metalloproteinase 9 (Fig. 5d) were upregulated at the site of lice attachment in the Copx2 group only. Two of the investigated genes had lower transcription at the site of lice attachment, namely collagen 10-*α* in the Cop, Copcop and Aducop groups (Fig. 5b) and inducible nitric oxide synthase in the Cop and Copcop groups (Fig. 5m). Collagen 10-*α* had also lower transcription at non-lice sites in the Copcop group, which also was the group with highest reduction at the site of lice attachment. Transcription of fibronectin precursor, cluster of differentiation 8, cluster of differentiation 4, immunoglobulin M, immunoglobulin T, interleukin 10, tumour necrosis factor-*α* and transforming growth factor-*β* did not differ between any treatment (Supplementary Table 1).

At the pre-adult stage, an upregulation in transcription of interleukin 8 (Fig. 5l), serum amyloid A (Fig. 5n), complement component 3 (Fig. 5g), tumour necrosis factor-*α* (Fig. 5o) and cathelicidin 2 (Fig. 5e) was evident at the site of lice attachment in all treatment groups with the smallest upregulation in the Copcop group. Tumour necrosis factor-*α* was also upregulated at non-lice sites in the skin in the Copcop group, while transcription of cathelicidin 2 was higher in lice-negative samples in the Copx2 group only. Interleukin 1-*β* (Fig. 5a), interleukin 2 (Fig. 5j), fibronectin precursor (Fig. 5c) and matrix metalloproteinase 9 (Fig. 5d) were significantly higher at the site of lice attachment in the Cop, Copx2 and Aducop groups. Whereas transcription of interleukin 10 was higher at the non-lice site in the Copx2 and Aducop groups (Fig. 5i). There was a downregulation of collagen 10-*α* (Fig. 5b) and cluster of differentiation 8 (Fig. 5f) at the site of lice attachment for all treatments. Immunoglobulin T was also downregulated at the site of lice attachment, but only in naïve fish (Cop and Copx2 groups) (Fig. 5h), while it was upregulated at the non-lice sites in the Copcop group. Aberrant expression of inducible nitric oxide synthase was observed in previously lice-infected fish, with a downregulation at the site of lice attachment in the Copcop group and an upregulation in non-lice sites in the Aducop group (Fig. 5m). Transcription of cluster of differentiation 4, immunoglobulin M, interleukin 4 and transforming growth factor-*β* did not differ between any treatment (Supplementary Table 1).

At the adult stage, transcription of several genes increased at the site of lice attachment in all treatment groups; this included interleukin 1-*β* (Fig. 5a), interleukin 8 (Fig. 5l), matrix metalloproteinase 9 (Fig. 5d), serum amyloid A (Fig. 5n) and tumour necrosis factor-*α* (Fig. 5o). Transcription of cathelicidin 2 (Fig. 5e) and complement component 3 (Fig. 5g) was also higher at the site of lice attachment for all treatment groups. Additionally, transcription was higher in lice-negative samples at the Copx2 group for cathelicidin 2 and in the Copx2 and Copcop groups for complement component 3. Interestingly, the lowest transcription levels for all these transcripts were observed in the Copcop group, while the expression of serum amyloid A, matrix metalloproteinase 9, cathelicidin 2 and complement component 3 was highest in the Copx2 group; it was highest in the Aducop group for interleukin 1-*β* and tumour necrosis factor-*α*. Fibronectin precursor (Fig. 5c) and transforming growth factor-*β* (Fig. 5p) had significantly higher transcription at the site of lice attachment both in the Copx2 and Aducop groups, while interleukin 4 (Fig. 5k) was only significantly increased in the Aducop group. Two transcripts were downregulated in the skin at the site of lice attachment in all treatments; this included cluster of differentiation 8 (Fig. 5f) and collagen 10-*α* (Fig. 5b). Collagen 10-*α* additionally showed increased transcription at non-lice sites in the Copcop group and reduced transcription in Copx2 group. Whereas interleukin 10 (Fig. 5i) and inducible nitric oxide synthase (Fig. 5m) were downregulated only at the site of lice attachment in the Copcop group. Lastly, the expression of immunoglobulin T was reduced at the site of lice attachment in both the Cop and Aducop groups (Fig. 5h). Transcription of cluster of differentiation 4, immunoglobulin M and interleukin 2 did not differ between any treatment (Supplementary Table 1).

## Discussion

The present study comparing salmon louse infection in fish with different infection history demonstrates that lice success on the host was lower in previously infected than in naïve fish. In nature, both wild and farmed salmonids are repeatedly exposed to and infected with salmon lice copepodids, resembling the treatment given to the Copcop group in this study. The specific growth rate of the host was negatively affected at high lice intensities and was lowest in fish previously infected with copepodids early in the infection. Contrarily, growth later in the infection was higher in fish previously infected with copepodids than in fish previously infected with adult lice and in naïve fish infected with a double dose of copepodids indicating compensatory growth. Similarly, stress (i.e. plasma cortisol levels) was higher early, but lower late in the infection in fish previously infected with copepodids. Parallel monitoring of transcriptional changes showed a stronger response early and an attenuated response later at the site of lice attachment in fish previously infected with copepodids.

### Parasite success

Results reported here suggest that already infected Atlantic salmon are less susceptible to infections. This is not in agreement with findings by Ugelvik *et al*. (2017*a*), who reported that already infected fish were more susceptible to new infections. Differences in experimental design could possibly explain the discrepancy. In the present study, fish were infected with conspecifics exposed to the same experimental treatment, while an earlier study used a common garden setup during the infection. Ugelvik *et al*. (2017*a*) reported significantly more lice from the second infection in the group carrying adult lice than in naïve controls. However, there was no difference in lice load between the control group and the group in which the louse from the previous infection was removed prior to the second infection (Ugelvik *et al*., 2017*a*). This could be caused by continuous immunosuppression by the louse in the fish maintaining adult lice from earlier exposures or it could be caused by preferential settlement of copepodids on hosts carrying adult lice when given the choice. Such an adaptive response at low lice intensities would increase the probability of the copepodid infecting a fish already carrying lice of the opposite sex. Previous studies have suggested that lice are able to detect and respond to cues from conspecifics (Morefield and Hamlin, 2022), for instance copepodids are more likely to settle on naïve than Atlantic salmon carrying lice at the chalimus II stage (O'Shea, 2005). However, this could not explain why fish carrying adult lice have higher lice loads than naïve fish when infected in the same infection tank (Ugelvik *et al*., 2017*a*). Possibly, copepodids respond differently to the presence of chalimus II than to adult lice. If the main driver of the number of lice acquired during the second infection is parasite-induced immunosuppression by the parasite, the same pattern should be observed when infection of naïve and already infected fish is performed in separate tanks. This was not the case here; contrarily, naïve fish had higher lice load than those carrying lice from earlier infections. Hence, the discrepancy is most readily explained by copepodids preferring to settle on already infected fish. However, it should be noted that both these studies were performed in tanks where copepodids were exposed to multiple hosts possibly creating a highly artificial infection environment, including multiple pressure waves and chemical cues demonstrated to affect copepodids (Mordue Luntz and Birkett, 2009).

Moreover, host immunosuppression by adult lice is not in agreement with the immune response reported here. We predicted that if the louse were immunosuppressing host responses, suppression should increase with the number of lice and the duration of the infection. Contrarily, an increased response in already infected fish was observed early in the infection and the largest response was observed in fish previously infected with adult lice. Additionally, the response was stronger in naïve fish infected with a double compared to a single dose of copepodids. A stronger immunological response in previously infected fish compared to naïve fish is in consonance with higher lice success in the latter group. Hence, if salmon lice are modulating host immune responses, it does not result in increased susceptibility to new lice infections.

### Lice effect on host weight and stress response

Cortisol levels were affected by treatment with different responses as the infection developed. At the chalimus stage, cortisol levels appeared to be dependent on time with the highest cortisol levels found in fish with the longest ongoing infection (Copcop group). However, later at the adult stage, an opposite pattern was observed with the lowest cortisol levels in the Copcop group and the highest levels in fish infected once with a double dose of copepodids. This is surprising since fish in these 2 groups carried similar lice loads and in the former the infection had lasted twice as long. This could indicate that the stress response in infected fish is attenuated after long-term exposure to the parasite and imply that fish over time are able to compensate for the negative effects of the lice infection, at least at the lice intensities studied here. Increased stress levels and pathogenicity are associated with development to the pre-adult stage (Grimnes and Jakobsen, 1996), suggesting that virulence depends on the development stage of the parasite or the duration of infection. The effect of cortisol on immune responses is difficult to predict since acute stress in fish is immunostimulatory, while chronic stress is considered immunosuppressive (Tort, 2011). Higher levels of cortisol in fish with high lice loads could increase susceptibility to new salmon lice infections and other pathogens due to the role of cortisol as an immunosuppressant (Pickering and Pottinger, 1989; Johnson and Albright, 1992)

At the chalimus stage, the growth rate of the salmon was reduced with an increasing number of lice and it was lower in fish previously infected with copepodids compared to fish infected once with a double dose of copepodids. Hence, salmon lice negatively affected the growth rate of Atlantic salmon early in the infection and unsurprisingly the effect was more pronounced in the group with the longest ongoing infection. Reduced specific growth rate with increasing lice loads has also previously been reported from other studies on Atlantic salmon in the laboratory (Fjelldal *et al*., 2020) and a negative correlation between body condition and lice density is also found in Atlantic salmon in the wild (Susdorf *et al*., 2018). A negative effect of lice on the growth rate at the adult stage is also supported by lower weight gain in naïve fish infected with a double compared to a single dose. Interestingly, the growth rate measured when lice had reached the adult stage was higher in fish infected twice with copepodids than in the groups previously infected with adult lice and naïve fish infected with a double dose of copepodids. The mean total number of lice per fish in the Copcop and Copx2 groups was similar and could indicate an ability of the fish to later compensate for the reduction in growth rate observed earlier in the infection. That fish are able to compensate for earlier suboptimal conditions is in agreement with studies showing that fish earlier exposed to restricted feeding compensate the weight loss by having higher growth rate than conspecifics that have been continuously fed, when conditions become favourable (Ali *et al*., 2003; Hvas *et al*., 2022).

### Transcriptional responses in the skin of lice infected salmon

The transcription of selected immune and would healing genes used here is previously associated with host responses towards salmon lice (Table 1). Most differential gene expression was observed in the skin at the site of lice attachment, while much fewer transcriptional changes were observed at non-lice sites. Hence, lice infection tended to result in more local than systemic immune responses in the skin of salmonids, confirming earlier findings (Øvergård *et al*., 2018; Dalvin *et al*., 2020; Ugelvik *et al*., 2022). Considering that salmon lice are ectoparasites in contact with the host only at the attachment site is unsurprising. Stronger local proinflammatory responses towards ectoparasites are also reported in 3 striped trumpeter (*Latris lineata*) and in carp (*Cyprinius carpio*) (Gonzalez *et al*., 2007; Covello *et al*., 2009). In our study, skin samples were consistently taken from the flank of the fish, since a previous study has reported variation in the expression of immune genes in different parts of the skin in Atlantic salmon (Holm *et al*., 2017). Hence, the transcriptional changes in the skin reported here might not reflect responses in scaleless and fin skin in the fish. Lice at the pre-adult and adult stages are mobile on the host, with especially males moving around there is a possibility that some samples were misidentified as either lice attachment or non-lice sites. However, that we observed differential expression between lice attachment and non-lice sites suggests that most samples were correctly identified. Transcription in the different lice treatments also varied during the infection, which may be caused by the progression of the different developmental stage of the louse or simply be a factor of time. Most noticeable was an early local increased expression of transcripts in fish that had previously been infected with copepodids, including transcripts involved in proinflammatory responses. This pattern was however not evident later at the pre-adult stage, where contrarily transcription levels of several transcripts were higher at the site of lice attachment in the other groups, but not in the group previously infected with copepodids. This was corroborated by lower transcriptional changes in fish infected with copepodids twice. Similarly, at the adult stage, several transcripts were upregulated for all treatments, but levels were lower in the group previously infected with copepodids. Overall, the expression of the investigated transcripts was lower in naïve fish infected with a single dose than those infected with either a double dose of copepodids or that previously was infected with adult lice, implying an effect of both parasite intensity and previous exposure on host responses towards the louse. This suggests that in fish already infected with copepodids, the response towards new lice infections is faster and stronger early but reduced later in the infection. This is congruent with the observed higher lice loss from the chalimus to the pre-adult stage in previously infected fish and could also explain why lice success is higher in the group infected with a single dose of copepodids than in previously infected fish.

Proinflammatory cytokines enhance inflammation at the site of infection, which can be an important part of host responses towards pathogens and parasites. Transcription of tumour necrosis factor-*α* in the skin observed in this study was increased at the site of lice attachment in all treatments, indicating a role in host responses towards the louse independent of previous exposure to the parasite. In addition, at the pre-adult stage, the mRNA level of tumour necrosis factor-*α* was higher at non-lice sites in the previously infected group, which was one of very few could imply a more systemic response in the skin. The presence of proinflammatory activity locally at the site of lice attachment was also supported by enhanced levels of interleukin 1-*β*. Interestingly, while mRNA levels of interleukin 1-*β* were highest at the chalimus stage sampling in the group previously infected with copepodids, it was lowest in this group at the adult stage, which implies a reduction in proinflammatory responses late in the infection in this group. Such a reduction is supported by transcription of interleukin 8. Interleukin 8 was induced at the site of lice attachment in all treatments, but again the lowest mRNA levels were observed in the group previously infected with copepodids.

Acute-phase proteins are also an important part of the early response to infections and increased transcription of serum amyloid A was evident in all treatments. However, late in the infection (pre-adult and adult stage) and congruent with the expression of proinflammatory cytokines, the lowest upregulation was seen in fish previously infected with copepodids. High levels of serum amyloid mRNA at the site of lice attachment corroborate the role of this gene in responses towards salmon lice (Braden *et al*., 2015) and the lower levels in group infected twice with copepodids could imply that the response is attenuated when the infection is long lasting. The antimicrobial peptide cathelicidin 2 was upregulated underneath the louse at all stages for all treatments, and in the single-dose group, it was also induced at non-lice sites at both the pre-adult and adult stages, which could imply systemic activation in the skin in this group. Interestingly, and in agreement with other investigated proinflammatory genes, the relative transcription of cathelicidin 2 at the site of lice attachment was highest at the chalimus and lowest at the pre-adult and adult stages in fish previously infected with copepodids. Demonstrating earlier induction of proinflammatory responses in this group, the response is diminished compared to the other treatments later in the infection. The concurrent upregulation of both cathelicidin 2 and interleukin 8 corroborates the presence of proinflammatory responses at the site of lice attachment.

Excessive inflammatory responses could lead to unnecessary damage to host tissues; hence, efficient responses towards parasites also depend on regulatory and anti-inflammatory responses. The regulatory cytokine interleukin 2 was upregulated at the site of lice attachment in all treatments. Transforming growth factor-*β* could increase inflammatory responses early in an infection and later suppress inflammation and it is also important in regulating extracellular matrix (Ignotz and Massagué, 1986; Qi *et al*., 2016). Upregulation of transforming growth factor-*β* in lice-infected fish is in agreement with previous findings in lice-damaged skin in both Atlantic (Skugor *et al*., 2008), Coho and Sockeye salmon (Braden *et al*., 2015) and a role in pathogen resistance in fish is also reported for both striped bass and trout (Rebl and Goldammer, 2018). Interestingly, there was a significant upregulation at the site of lice attachment only in groups with the highest proinflammatory responses late in the infection (Copx2 and Aducop), which could also induce stronger regulatory and anti-inflammatory responses. This is supported by higher upregulation of the regulatory cytokine interleukin 10 at non-lice sites.

The mRNA levels of cluster of differentiation 8 were reduced at the site of lice attachment at the pre-adult and adult stages, but no effect of treatment was evident in transcription of this gene. The reduced transcription of cluster of differentiation 8 could indicate fewer cytotoxic cells at the site of lice attachment, which could increase susceptibility to viral infections (Braden *et al*., 2015). Additionally, lice-infected fish are also more prone to secondary infections due to damages inflicted on the skin by the grazing activity of the parasite, and together with the reduction in cytotoxic cells, this could explain the higher susceptibility to infectious salmon anaemia virus observed in infected fish (Barker *et al*., 2019).

### Wound healing

By feeding on skin, mucus and blood of its host, salmon lice, especially at the mobile stages, cause skin damage. To reduce susceptibility to secondary infection, it is important for the host to rapidly repair the damaged skin. Matrix metalloproteinase 9 was upregulated at the site of lice attachment at the adult stage for all treatments. Contrastingly, at the chalimus stage, mRNA levels of collagen 10-*α* were reduced at the site of lice attachment. Additionally, aberrant expression of collagen 10-*α* at non-lice sites between the Copcop and Copx2 at the adult stage could indicate systemic responses at high lice intensities, and moreover suggests that the response depends on the duration of the lice infection. Aberrant expression of wound healing transcripts at the site of lice attachment could affect the ability of the host to repair lice-inflicted damage, which could affect the pathology induced by the ectoparasite.

## Conclusion

The salmon louse is a major pest in Atlantic salmon aquaculture and a threat to wild salmonids. Knowledge on how previous exposure affects host responses towards the parasite and the ability of the louse to infect and survive on the host presented herein is therefore of importance to both the industry and regulatory authorities. Transcriptional responses in the skin towards the louse varied with previous exposure to the parasite, but most changes were observed locally at the site of lice attachment. To be able to breed more lice-resistant salmon in the future, it would be beneficial to have stronger adaptive immune responses in the whole skin or in systemic immune organs.

## Supporting information

Stølen Ugelvik et al. supplementary material Stølen Ugelvik et al. supplementary material

## Data Availability

Data are available as supplementary material.

## Supplementary material

The supplementary material for this article can be found at https://doi.org/10.1017/S0031182023000847
