# Supplementary material for: Repeated exposure affects susceptibility and responses of Atlantic salmon (Salmo salar) towards the ectoparasitic salmon lice (Lepeophtheirus salmonis)
Source: Parasitology. 2023 Sep 14;150(11):990–1005. doi: 10.1017/S0031182023000847 (PMC10941223; doi:10.1017/S0031182023000847)
Supplement: Stølen Ugelvik et al. supplementary material [file S0031182023000847sup001.docx]

**Supplementary Material Table 1.** Mean fold change (±SE), p- and t-values depending on treatment (copx2= infected once with a double dose of copepodids; copcop infected twice with singles doses of copepodids; aducop infected with adult lice and later with a single dose of copepodids) and lice development stage (chalimus II, pre-adult I and adult stage) for the investigated immune and wound healing transcripts relative to negative samples from the group infested once with a single dose of copepodids (cop group). For each fish two skin samples were taken, one directly underneath the louse (positive sample) and one from a similar location without lice (negative sample).

**Supplementary Material Table 2.** Total number of lice (lice previosuly established on the fish and lice from the infection), mean number of lice (±SE) and infection success and survival (%) at the infection depending on treatment (Cop, Copx2, Copcop and Aducop) at three louse development stages (chalimus, pre-adult and adult).

| Total number of lice | Infection | | Treatment | Stage |
| --- | --- | --- | --- | --- |
| Mean number of lice (±SE) | Mean number of lice (±SE) | Infection success and survival (%) (±SE) |  |  |
| 41.7 (3.0) | 41.7 (3.0) | 69.5 (6.4) | Cop | Chalimus |
| 64.9 (5.3) | 64.9 (5.3) | 54.1 (4.4) | Copx2 |  |
| 62.5 (2.7) | 38.9 (2.5) | 64.8 (4.2) | Copcop |  |
| 39.1 (2.9) | 37.1 (3.0) | 61.8 (5.0) | Aducop |  |
| 38.6 (3.0) | 38.6 (3.0) | 64.3 (5.2) | Cop | Pre-adult |
| 67.0 (2.5) | 67.0 (2.5) | 55.9 (2.1) | Copx2 |  |
| 52.9 (1.9) | 31.9 (1.5) | 53.2 (2.5) | Copcop |  |
| 29.2 (2.1) | 28.2 (2.2) | 46.9 (3.6) | Aducop |  |
| 29.1 (2.5) | NA | NA | Cop | Adult |
| 47.1 (3.3) |  |  | Copx2 |  |
| 45.5 (2.6) |  |  | Copcop |  |
| 18.9 (1.4) |  |  | Aducop |  |
